# Optimal formulations of local foods to achieve nutritional adequacy for 6–23-month-old rural Tanzanian children

**DOI:** 10.1080/16546628.2017.1358035

**Published:** 2017-07-30

**Authors:** Jofrey Raymond, Neema Kassim, Jerman W. Rose, Morris Agaba

**Affiliations:** ^a^ School of Life Science and Bioengineering, Nelson Mandela African Institution of Science and Technology (NM-AIST), Arusha, Tanzania; ^b^ SolBridge International School of Business, Woosong University, Daejeon, Republic of Korea

**Keywords:** Optimal food formulations, local foods, infants and young children, linear programming, Tanzania

## Abstract

**Background**: Achieving nutritional goals of infants and young children while maintaining the intake of local and culture-specific foods can be a daunting task. Diet optimisation using linear goal programming (LP) can effectively generate optimal formulations incorporating local and culturally acceptable foods.

**Objective**: The primary objective of this study was to determine whether a realistic and affordable diet that achieves dietary recommended intakes (DRIs) for 22 selected nutrients can be formulated for rural 6–23-month-old children in Tanzania.

**Design**: Dietary intakes of 400 children aged 6–23 months were assessed using a weighed dietary record (WDR), 24-hour dietary recalls and a 7-days food record. A market survey was also carried out to estimate the cost per 100 g of edible portion of foods that are commonly consumed in the study area. Dietary and market survey data were then used to define LP model parameters for diet optimisation. All LP analyses were done using linear program solver (LiPS) version 1.9.4 to generate optimal food formulations.

**Results**: Optimal formulations that achieved DRIs for 20 nutrients for children aged 6–11 months and all selected nutrients for children aged 12–23 months were successfully developed at a twofold cost of the observed food purchase across age groups. Optimal formulations contained a mixture of ingredients such as wholegrain cereals, Irish potatoes, pulses and seeds, fish and poultry meat as well as fruits and vegetables that can be sourced locally.

**Conclusions**: Our findings revealed that given the available food choices, it is possible to develop optimal formulations that can improve dietary adequacy for rural 6–23-month-old children if food budget for the child’s diets is doubled. These findings suggest the need for setting alternative interventions which can help households increase access to nutrient-dense foods that can fill the identified nutrient gaps.

## Introduction

Adequate nutrition during the first 1000 days, beginning at conception and extending through to the second birthday of a child, is a critical window for preventing undernutrition and its long-term consequences [1–3]. Poor feeding practices during this vulnerable period can increase the risk of undernutrition, morbidity and mortality of infants and young children. Early-childhood undernutrition can impair the physical and cognitive development of a child and can also weaken the ability of the child to fight against deadly infectious diseases [4]. In the long term, early childhood undernutrition can lead to reduced school performance, lower economic productivity, shorter adult stature and decreased offspring birth weight [2,4–6].

Most households in Sub-Saharan Africa (SSA) do not have adequate nutrition knowledge for decision making that accounts for the full cost–benefit analysis of a balanced diet. Feeding practices in typical African households are mostly geared to abetting hunger as a singularity [7], and so nutrition is rarely considered or factored into food security strategies. Furthermore, the perception that nutrient-rich meals are expensive, coupled with traditional notions of what constitutes food (‘food is maize’), and missed opportunities around other foodstuffs contributes to undernutrition, especially, among low-income urban and most rural households [8]. As a result, in SSA, more than 38.5% (>56 million) children are stunted, 19.6% (30 million) are underweight and 9.0% (15 million) are wasted. Similarly, deficiencies of essential micronutrients such as vitamins A, folic acid, iron, iodine, calcium and zinc are extensive and have devastating impacts [9].

In Tanzania particularly, more than one-third (35.5%) of children under 2 years of age are facing chronic undernutrition [10]. Micronutrient deficiencies among children are common, and prevalence of anaemia, iron and vitamin A deficiency among children aged 6–23 months are 73%, 42% and 33%, respectively [11]. Poor infant and young child feeding (IYCF) practices are among the main causes of the prevalent childhood malnutrition in the country. The analysis of data from Tanzania Demographic Health Survey (TDHS) showed that sugary foods (13%) are more frequently consumed than micronutrient-rich foods such as fortified infant cereals (4%), eggs (8%) and vitamin-A-rich fruits (0%) [12]. This evidence suggests a general need to improve IYCF practices in SSA communities. The use of locally available nutrient-dense foods can sustainably improve complementary feeding practices in a target population [13]. The linear programming (LP) approach can facilitate the optimal use of locally available foods to improve the dietary adequacy of vulnerable populations [14]. The LP approach needs to be carried out based on local availability and accessibility of nutrient-dense foods in the target population [15].

Several studies have reported mixed results on the ability of local foods to meet nutrient requirements for children in low-income countries. One study in Kenya, for instance, showed that LP modelled diets from local foods could achieve nutrient requirements for young children aged 12–23 months [16]. In contrast, the same Kenyan study revealed that local foods could not meet micronutrient requirements for infants aged 6–11 months even after integrating wild foods to the LP model [16]. Another study in Mozambique showed that optimal combinations of locally available foods can meet all micronutrient recommendations for young children aged 12–36 months [17]. However, the Mozambique’s study used EARs instead of RNIs in the LP models, meaning that the selected diet would not meet the requirements of 50% of the population for those nutrients at 100% of the EAR. Two recent studies in Kenya that used RNIs in LP models showed that it is difficult to achieve nutrient requirements for infants and young children using locally available foods only [15,18]. Despite the mixed results from previous and recent studies, the LP has proven useful in identifying the cheapest possible combination of food ingredients that meet a set of nutritional requirements in low-income communities [19]. The LP tool has also proven useful in predicting limiting nutrients [20] and assessing the cost-effectiveness and economic value of food fortification in a population [21]. The LP can, therefore, help practitioners to develop realistic and affordable formulations in resource-poor communities. The primary objective of the present study was to determine whether a realistic and affordable diet that achieves dietary recommended intakes (DRIs) for selected nutrients can be formulated for rural 6–23-month-old children in Tanzania.

## Methods

### Study site

The study was conducted in six randomly selected villages out of 59 in Bahi District, the central zone of Tanzania. This district was purposively selected because it is located in the region with high prevalence of stunting among infants and young children under two years of age [22].

### Study design

A cross-sectional survey was conducted from September to December 2015 during the dry season to assess dietary consumption patterns of 400 children aged 6–23 months. A market survey was also carried out to determine the cost per 100 g of edible portion of local foods commonly consumed in the study population. Dietary and market survey data were then used to define LP model parameters for diet optimisation. All the analyses were done using MS Excel 2013 and linear program solver version (LiPS) 1.9.4 to generate an optimal solution. Additionally, anthropometric z scores for infants and young children were calculated based on WHO’s 2006 Child Growth Standards [23] with the use of ProPAN software (version 2.0). A child was categorised as wasted, underweight or stunted if his or her z-scores for weight-for-length, weight-for-age or length-for-age were less than −2 SD.

#### Subjects and sampling

Six villages were randomly selected from 59 villages in Bahi District and 67 children were randomly selected from each village [24]. The inclusion criteria were that a child was 6–23 months of age inclusive and that his or her primary caregiver was available for and agreed to participate in the study survey. Moreover, the child was supposed to be healthy and not suffering from any disability that may affect his or her dietary intakes. If more than one child in a household met the inclusion criteria, then one was randomly selected.

#### Food consumption pattern

Dietary data were collected using 24-hour dietary recalls, weighed dietary record (WDR) and 7 days food records described in previous studies [24–26]. The WDR method was used to collect data on food consumption for 7 days; 24-hour dietary recalls and 7-day food records were used to describe food patterns in 7 days. Food portion sizes were not collected for the 24-hour recall and 7-day food records.

A single 24-hour recall was done to obtain information on foods and beverages consumed within 24 hours before the WDR day. During the interview, mothers were asked to recall all foods and beverages consumed by their infants in the past 24 hours. To help identify foods consumed, pictures and food models were shown to a mother or a caregiver so that she or he could point out the food items. Mothers were also asked to mention other foods and beverages that were not included in our food models and pictures.

In the WDR method, all foods and beverages consumed by a child were weighed using an electronic kitchen scale (CAMRY, Model EK3131, precision ±2 g) and recorded. A researcher was present at the household for 12 hours of the day to observe and weigh the amount of all foods and beverages served and consumed by the child as well as leftovers. A 12-h recall for all foods and beverages consumed after the observer has left the household was also collected. Then, mothers were asked to estimate the amount of recalled foods in local cups or utensils, and the estimated amount was then weighed using the real food models. All composite foods and dishes were broken down into their individual ingredients such as added oil, baobab powder, sugar, salt, tomato, carrots, onions, and so forth. WDR were collected on all days of the week to account for the effect of each day of the week on dietary intakes of the subjects.

In the case of 7-days food records, a self-administered 7-day food tally was done to obtain information on the frequency of foods and beverages consumed after the WDR day. In this approach, the primary caregivers were asked to record all foods and beverages consumed by their children during the 7-day period. For the illiterate primary caregivers and those who forgot to record foods consumed, a 12-hour recall was then performed.

#### Market survey

A market survey was also conducted in one local market, which was often used by mothers in each village. Only one price per food item was collected in small shops available in each village. If the survey location had a market with multiple vendors, three different prices per food item were collected, and an attempt was made to include both the highest and lowest prices across all vendors. For cooked composite dishes, each raw ingredient was weighed and their costs were summed to obtain the cost for all ingredients. The final cooked food was also weighed, and the cost per 100 g of cooked food or composite dish was then estimated. The cost was linked to the food composition database for our LP models.

#### Preparation of model parameters

Data from the dietary assessment survey defined the model parameters. The preparation of LP model parameters was done in Microsoft Excel 2013. These parameters included food subgroups, a median serving size for each food subgroup, the lower, median, and upper limits of serving sizes, and the maximum number of servings per week for food groups and food subgroups. Food subgroups were defined by grams (serving sizes) of individual foods that were consumed by ≥5% of children and nutrient-dense foods consumed by ≤5%. The serving size for each food subgroup was defined by the median serving size for children who consumed the food in each age group. The lower, median and upper limits of serving sizes were defined as 10th, the 50th and 90th percentile of food patterns for all infants and young children in each age group. The maximum number of servings per week for optimal food patterns was defined as 1, 2, 3, 4, 5, 6 or 7 when 0–5%, 6–12%, 13–22%, 23–34%, 35–47%, 48–65% and 66–100%, respectively, of the children consumed the food [26]. These LP parameters were used to set up the LP models for the analyses in LiPS software version 1.9.4. The diets were modelled for a 7-day period.

#### Food composition database

Dietary intakes of nutrients were estimated based on Tanzania Food Composition Tables (TFCT) [27] and United States Department of Agriculture Food Composition Database (USDA) [28]. In some cases, the amount of foods served and consumed were converted to their raw form using cooked to raw conversion factors so as to match nutrient values in food composition databases [24]. An average of 33 food items appeared in the dietary records and were categorised into seven major food groups and 11 food subgroups based on the WHO food groups and culinary usage for both breastfed and non-breastfed infants and young children aged 6–23 months [29]. The seven major food groups included: grains, roots and tubers; legumes, nuts and seeds; dairy products; flesh foods; eggs; vitamin-A rich fruits and vegetables, other fruits and vegetables; and a miscellaneous group. The nutrient profiles were created based on 7-days weighed intakes using median proportion weights per food item. In order to avoid overestimation, nutrient values for the foods consumed in cooked state were adjusted for cooking losses using USDA retention factors [30]. Nutrient profiles were calculated separately for each age group and were used as input data for our LP models.

#### Objective function of linear programming models

The objective function was to minimise deviations from the population’s food sub-group patterns for 11 food sub-groups while simultaneously meeting the required dietary standards. Based on the study by Okubo et al. [31], the objective function is represented by:

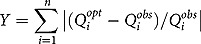

where Y denotes the objective function to minimise, and 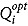 denotes the quantity (g) of food subgroup i in the optimised food intake pattern, and 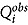 denotes the median quantity (g) of food subgroup i in the observed food intake pattern. The absolute value Y was nonlinear. In order to apply LP, we had to transform Y into a linear function using goal programming approach described in previous studies [19,31,32]. New decision variables ≥0, representing positive (P_1_ to P_33_) and negative (N_1_ to N_33_) deviation from the quantity of the observed food subgroup, were created and defined as follows:

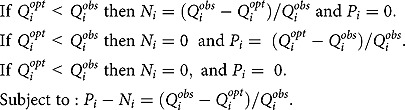

The new linear function termed Yʹ was expressed as the sum of the deviational variables and minimised as follows:

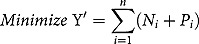

where *N_i_* and *P_i_* are negative and positive deviational variables representing deviations from the *i*^*th*^ goal.

Each food subgroup in the objective function was linked to the nutrient profile and cost databases established for the present study. The model calculated the intake of food subgroups at all times and checked whether nutritional constraints were satisfied. The observed intake of each food subgroup was defined according to the median intake of that food subgroup across the whole population.

#### Nutritional constraints for linear programming models

We introduced a set of constraints to our LP models to minimise the gap between observed and optimised food intake patterns. The intention was to ensure that the solution selected by a model satisfies each of the specified constraints. Food use limits were set to ensure that program models did not exceed the amount of foods that is usually consumed by the target population. These limits were derived from the actual intake patterns for each age group as reported in a 12-hour WDR. The dietary intake of all foods from each food subgroup was required not to exceed the 90th percentile of intake for each age group. Food group and sub-group constraints were defined by the grams of individual foods in each age group. Food intakes were constrained within the range from the 10th percentile to 90th percentile of the observed intakes for each age group [31]. In the present study, however, the intake of small dried fish (sardines), meat, vegetables, fruits and seeds was very low. For these foods, we, therefore, had to exceptionally set a maximum intake level to ≥90th percentile for each age group aiming to promote consumption of these nutrient-dense foods.

Nutritional constraints were included to ensure that the nutritional content of each optimised food intake pattern was equal to or greater than the desired value, which was based on the DRIs for infants and young children. DRIs for the selected nutrients such as protein, calcium, iron, zinc, copper, magnesium, manganese, phosphorus, potassium, sodium, vitamin A (RE), folate, vitamin B_1_, vitamin B_2_ niacin, vitamin B_6_, vitamin B_12_, vitamin C, vitamin D, vitamin E and pantothenic acid published by the World Health Organization (WHO) for infants and young children [33] was the criterion for determining whether each nutritional goal had been achieved by the optimised food intake pattern. Our LP model assumed low bioavailability of iron and zinc. In some cases, tolerable upper intake level (UL) was also used to create acceptable nutrient ranges. Energy constraint for moderately breastfed children was also included in the model to ensure that the energy content of the optimised food intake pattern was equal to the estimated energy requirement (EER) for each age group. The energy and nutrient contribution from breast milk was taken into account to ensure that the realistic diets were selected. Since the mean breast-milk intakes for the study population were unknown, we, therefore, used the published WHO average breast milk intakes of 660 g/d, 616 g/d and 549 g/d for children aged 6–8 months, 9–11 months and 12–23 months, respectively, in our LP models [34]. In addition, the cost constraint was set to ensure that the cost of the optimal formulation was equal to the maximum cost of all diets consumed by the children in each age group.

## Results

### Characteristics of subjects

Four hundred infants and young children from 400 predominantly rural households were surveyed in Bahi District in Dodoma Region of central Tanzania. Among the 400 subjects, 50% were children aged 12–23 months, 25% were infants aged 9–11 months and 25% were infants aged 6–8 months. Overall, 40% of children in the study area were facing chronic undernutrition, i.e. their height-for-age z-scores were less than −2 SD (Table 1). All infants and young children in the present study were still being breastfed at the time of the survey.

**Table 1.** Socio-demographic characteristics of the study population (n = 400 child–mother pairs).

Variable		Mean/Frequency	Std. Deviation/ (%)	Min, Max
Age	Age of the respondent	27	7.0^sd^	16,56
Head of household	If respondent is head of household	40	10.0	
Marital status	Married	330	82.5	
	Single	38	9.5	
	Widowed	10	2.5	
	Separated	22	5.5	
Maternal education	Illiterate	154	38.5	
	Read and write	8	2.0	
	Primary	200	50.0	
	Secondary	36	9.0	
	Tertiary	2	0.5	
Household size	Number of people living in the household	5	2.1^sd^	2,14
Child nutrition status (aged 6–23 months)	Height-for-age z-score < −2 SD	162	40.5	

### Observed food patterns

The total of 28, 32 and 42 food items were reported in the dietary data for children aged 6–8 months, 9–11 months and 12–23 months, respectively. The observed diets were mainly cereal-based, with few animal-source foods across all age groups. From the list of all foods, seven major food groups were formed based on the WHO food groups for both breastfed and non-breastfed infants and young children aged 6–23 months [29]. Main food groups that were formed include grains, roots and tubers; legumes, nuts and seeds; dairy products; flesh foods; eggs; vitamin-A rich fruits and vegetable; other fruits and vegetables; and a miscellaneous group. In addition, the 10th, 50th and 90th percentiles of the observed intake of all foods that could be successfully promoted were established across age groups (Table 2).

**Table 2.** Food group and sub-food group constraints included in linear programming models.

		6–8 months (*n* = 100)			9–11 months (*n* = 100)			12–23 months (*n* = 200)		
		Lower	Average^a^	Upper	Lower	Average^a^	Upper	Lower	Average^a^	Upper
Food group (g/day)	Subgroup (g/day)	P10	P50	P90	P10	P50	P90	P10	P50	P90
Grains, roots and tubers		37.3	98.1	152.4	65.7	118.6	290.8	106.6	195.1	387.2
	Whole grains, flour	15.3	58.4	112.7	31.9	61.6	147.3	31.2	85.1	157.1
	Refined grains, flour	4.0	9.7	9.7	3.8	12.0	23.6	35.4	55.0	68.6
	Irish potatoes, raw	18.0	30.0	30.0	30.0	45.0	120.0	13.0	20.0	106.5
	Sweet potatoes, raw	0.0	0.0	0.0	0.0	0.0	0.0	27.0	35.0	55.0
Legumes, nuts and seeds		3.6	13.0	46.5	5.4	12.3	60.4	17.8	27.7	65.8
	Seeds, dried, raw	0.7	1.0	25.5	1.2	2.0	36.0	1.7	2.4	28.5
	Pulses, dried, raw	3.0	12.0	21.0	4.2	10.3	24.4	16.1	25.4	37.3
Dairy products	Cow milk, raw	0.0	0.0	0.0	4.0	32.4	129.2	15.5	33.9	102.8
Flesh foods		3.8	5.0	31.3	5.6	7.2	35.8	7.0	11.5	58.7
	Meat	1.6	2.0	2.8	4.0	4.7	5.8	3.0	5.0	6.9
	Fish, dried	2.2	3.0	28.5	1.6	2.5	30.0	4.0	6.5	51.8
Eggs	Egg, boiled	0.0	0.0	0.0	13.7	18.7	23.6	17.0	25.0	65.0
Vitamin-A rich fruits and vegetables		88.8	104.0	228.2	71.4	106.8	224.2	70.6	127.9	348.2
	Fruits	0.9	2.2	5.8	23.9	34.9	58.7	36.3	38.3	55.7
	Green and yellow vegetables	61.1	74.7	191.3	36.9	59.5	145.8	21.5	53.5	237.5
Other fruits and vegetables		26.8	27.2	31.1	10.5	12.4	19.7	12.9	36.1	55.1
Others		2.8	5.9	17.3	2.8	7.6	15.9	2.9	6.9	16.6
	Fats and oils	0.3	1.5	6.0	0.3	1.0	3.0	0.3	1.2	4.8
	Salt	0.2	0.4	2.1	0.1	0.3	1.3	0.2	0.4	1.5
	Sugar	2.3	4.0	9.2	2.4	6.3	11.7	2.4	5.3	10.4

^a^Average serving sizes were based on the number of observed median servings of food in each food subgroup.

### Optimal food intake patterns

Optimal food patterns were generated and the differences between observed and optimised food intake patterns were established (Table 3). In the present analysis, the difference between observed and optimal intakes of fruits was 3.6, 23.8 and 17.4 g for children aged 6–8, 9–11 and 12–23 months, respectively. Also, the difference between observed and optimal green and yellow vegetable intake was 116.6 g for children aged 6–8, 86.3 g for children aged 9–11 months and 184 g for children aged 12–23 months. Similarly, the difference between observed and optimised intakes of flesh foods (fish and meat) was 0.6 g for children aged 6–8 months, 28.7 g for children aged 9–11 months and 47.2 g for children aged 12–23 months. Furthermore, the difference of 24.5, 34.0 and 26.2 g for children aged 6–8, 9–11 and 12–23 months, respectively, was observed in oily seed intakes. Eventually, a total difference of 4.2, 4.9 and 4.2 g for children aged 6–8, 9–11 and 12–23 months respectively was observed in a miscellaneous food group such as fats/oil, salt and sugar.

**Table 3.** Comparison of food quantities (g/day) between observed and optimised food intakes.

		6–8 months (*n* = 100)		9–11 months (*n* = 100)		12–23 months (*n* = 200)	
Food group	Subgroup	Observed	Optimised	Observed	Optimised	Observed	Optimised
Grains, roots and tubers		98.1	33.3	118.6	61.6	195.1	85.1
	Whole grains, flour	58.4	15.3	61.6	61.6	85.1	85.1
	Refined grains, flour	9.7	0.0	12.0	0.0	55.0	0.0
	Irish potatoes, raw	30.0	18.0	45.0	0.0	20.0	0.0
	Sweet potatoes, raw	0.0	0.0	0.0	0.0	35.0	0.0
Legumes, nuts and seeds		13.0	46.5	12.3	41.3	27.7	43.9
	Seeds, dried, raw	1.0	25.5^a^	2.0	36.0^a^	2.4	28.5^a^
	Pulses, dried, raw	12.0	21.0^a^	10.3	5.3	25.4	15.4
Dairy products	Cow milk, raw	0.0	0.0	32.4	0.0	33.9	0.0
Breast milk	Human milk^b^	660.0	660.0	616.0	616.0	549.0	549.0
Flesh foods		5.0	5.6	7.2	35.8	11.5	58.7
	Meat, raw	2.0	2.8^a^	4.7	5.8^a^	5.0	6.9^a^
	Fish, dried	3.0	28.5^a^	2.5	30.0^a^	6.5	51.8^a^
Eggs	Egg, boiled	0.0	0.0	18.7	0.0	25.0	0.0
Vitamin-A rich fruits and vegetables		104.0	228.1	106.8	224.2	127.9	348.2
	Fruits	2.2	5.8^a^	34.9	58.7^a^	38.3	55.7^a^
	Green and yellow vegetables	74.7	191.3^a^	59.5	145.8^a^	53.5	237.5^a^
Other fruits and vegetables		27.2	31.1	12.4	19.7	36.1	55.1
Others		5.9	1.8	7.6	2.7	6.9	2.7
	Fats and oils	1.5	0.0	1.0	0.3	1.2	0.3
	Salt	0.4	0.0	0.3	0.0	0.4	0.0
	Sugar	4.0	1.8	6.3	2.4	5.3	2.4
Total cost TZS (USD)		500 (0.3)	1122 (0.6)	780 (0.4)	1335 (0.7)	1325 (0.7)	2650 (1.3)

^a^90th percentile upper limit constraint was reached for the food group and subgroup patterns.

^b^The amount of breast milk was estimated from values published by WHO [34].

### Nutrient profiles for observed and optimised diets

The nutrient profiles for observed and optimised food patterns were generated in each age group (Table 4). Based on the reference dietary intake values published by WHO for infants and young children [33,35,36], our results showed that the number of nutrients for which nutritional goals were not achieved in the observed food intake pattern was six for infants aged 6–8 months, three for infants aged 9–11 months, and three for children aged 12–23 months. The number of nutrients for which the nutritional goals were not achieved in the optimised diets was two for both children aged 6–8 and 9–11 months. Optimal formulation for children aged 12–23 months achieved the nutritional goal of DRIs for all 22 studied nutrients including energy, protein, calcium, iron, zinc, copper, magnesium, manganese, phosphorus, potassium, sodium, folate, niacin, vitamin A, vitamin B1, vitamin B12, vitamin B2, vitamin B6, vitamin C, vitamin D, vitamin E and pantothenic acid.

**Table 4.** Comparison of nutrient contents between observed and optimised daily food intake patterns.

	6–8 months (*n* = 100)		9–11 months (*n* = 100)		12–23 months (*n* = 200)	
Nutrients^c^	Observed^b^	Optimised	Observed^b^	Optimised	Observed^b^	Optimised
Energy (Kcal/day)	841.2	888.1	925.9	1043.9	1110.0	1079.7
Protein (g/day)	19.4	33.9	24.8	33.4	35.9	44.7
Calcium (mg/day)	319.8^a^	630.5	339.5^a^	685.9	395.7	726.1
Iron (mg/day)	4.8^a^	9.7^a^	6.1^a^	10.6^a^	7.0^a^	11.7
Zinc (mg/day)	3.0^a^	5.0	11.3	13.7	10.8	12.6
Copper (mg/day)	0.9	2.1	1.0	2.3	1.2	2.3
Magnesium (mg/day)	131.4	263.5	189.5	324.5	234.1	351.0
Manganese (mg/day)	3.2	5.5	2.1	3.6	5.2	7.6
Phosphorus (mg/day)	342.4^a^	570.8	445.1	634.4	609.4	788.5
Potassium (mg/day)	1021.3	1606.2	1361.0	1637.4	1440.4	1875.2
Sodium (g/day)^c^	296.7	161.6	294.5	173.2	706.4	367.2
Folate (μg/day)	134.3	228.2	118.9	179.8	162.8	215.2
Niacin (mgNE/day)	4.2	7.7	6.6	8.4	7.9^a^	10.3
Vitamin A (μgRE/day)	793.2	1336.3	792.9	1052.6	1289.0	1989.7
Vitamin B1 (mg/day)	0.7^a^	1.4	0.7	1.2	1.2	1.9
Vitamin B12 (μg/day)	0.4	0.7	0.6	0.7	0.9	1.3
Vitamin B2 (mg/day)	2.4	7.9	1.8	6.2	5.0	10.1
Vitamin B6 (mg/day)	0.5	0.9	0.7	1.0	0.8	1.2
Vitamin C (mg/day)	69.5	114.8	53.1	90.6	72.5	126.1
Vitamin D (μg/day)	1.0^a^	4.1^a^	3.6^a^	4.2^a^	4.4^a^	6.8
Vitamin E (mg/day)	2.5	5.5	2.3	3.4	5.1	7.5
Pantothenic acid (mg/day)	2.4	2.6	2.3	2.3	2.9	3.1

^a^Nutrients not meeting the dietary recommended intakes (DRIs).

^b^Observed intake of nutrients in each age group was based on the median population intake of nutrients.

^c^Amount of breast milk intake used to calculate energy and nutrient intakes was 660.0 (g/day), 616.0 (g/day) and 549.0 (g/day) for children aged 6–8 months, 9–11 months and 12–23 months, respectively [34].

## Discussion

Age-specific optimised food intake formulations that achieved a set of 20–22 nutrient recommendations were generated using LP models given the DRIs for infants and young children aged 6–23 months in developing countries. The present analysis demonstrates how nutrient-based recommendations can be turned into nutritionally adequate food intake patterns with minimal modification of current feeding practices among young children in low-income communities. Most of our optimised food intake patterns ranged within 10th and 90th percentile limits of the observed intake patterns at food group levels (Table 3), demonstrating that the achieved solution is within the cultural eating habits of the study population. In addition, the intake of some baseline nutrient-dense foods such as seeds, fish, meat, fruits and vegetables reached the upper limit (90th percentile). This finding indicates that the consumption of foods within these food groups needs to increase.

For easier interpretation of data, we assumed that dietary modification was required when the difference between observed and optimised food intake patterns was more than 10%. In the analysis, we observed that though nutritional constraints set by DRIs did not differ significantly between age groups, dietary modifications that were needed to achieve the generated nutritional goals differed according to age group. Fruits and vegetables, for example, needed to be increased by 119, 110 and 172% for children aged 6–8, 9–11 and 12–23 months, respectively. Also, foods of animal source such as fish and poultry meat needed to be increased by 526% for children aged 6–8 months, 401% for children aged 9–11 months, and 410% for children aged 12–23 months. Likewise, our analysis revealed that a 257, 236 and 58% increase of pulses and seeds were needed for children aged 6–8, 9–11 and 12–23 months, respectively. The present analysis demonstrated that meeting nutritional goals for the studied population requires a considerable increase in the consumption of nutrient-rich foods such as fruits, seeds and animal source foods for all age groups. These findings agree with reports from previous studies on the use of LP to improve the intake of problem nutrients among Myanmar and Kenyan children under two years of age [3,18]. Nutrient-based dietary guidelines for feeding Tanzanian children aged 6–23 months are not well documented, making it difficult to compare our modelled diets with those of other studies. The present analyses might, therefore, contribute to the improvement of dietary guidelines for infants and young children in low-income communities in Tanzania.

The optimised food intake patterns called for a marked reduction of refined grains to keep consumption of whole grains within at least 10th percentile limit in all age groups. The optimised models revealed a zero intake of salt so as to maintain the consumption of sugar and vegetable oil within at least 10th percentile across all age groups. Nevertheless, the gap between observed and optimised food intake patterns in children aged 6–8 months was to some extent higher than in other age groups. These results were slightly consistent with food choices recommended in Canada’s Food Guidelines [37]. However, the reasons for differences in the degree of dietary modification required for each age group are unclear, though they might be explained based on existing age-specific feeding practices. The observed intake of most nutrient-dense foods was relatively lower in children aged 6–8 months compared to older age groups (Table 3). Also, the energy requirement was relatively lower in infants aged 6–8 months compared to older groups. Thus, achieving nutritional goals for infants aged 6–8 months needed a substantial increase in nutrient-dense foods such as pulses and seeds, small dried fish, fruits and vegetables. Previous studies conducted in Kenya reported similar findings about the required dietary modification based on age group [15,18]. These two Kenyan studies revealed that achieving nutritional goals for infants and young children needs additional modification to local diets. Such interventions may include low-cost fortified foods, increased access to animal-source foods or home fortification strategies using micronutrient powders.

Our present analysis showed that about 70% of all studied nutrients met the RDI using the observed food patterns. The number of nutrients that achieved recommended intakes reached 91% for children aged 6–11 months and 100% for children aged 12–23 months after using diet optimisation models. This indicates that our modelled diets from local foods could improve the nutritional quality of complementary foods for rural Tanzanian children aged 6–23 months. However, it should be noted that although our optimal modelled diets achieved nutritional goals for children aged 12–23 months, the optimal formulation contained exactly 100% RDIs of iron and niacin, indicating that these are limiting nutrients in the studied population. Also, one should note that modelled diets achieved only 50% RDI for iron in infants and young children aged 6–8 months and 9–11 months, suggesting that iron is the problem nutrient in a population. Iron deficiency during 6–23 months of age has been associated with impaired cognitive performance and other irreversible potential lifelong behaviour changes which can limit a child’s ability to benefit from economic opportunities in adulthood [38]. This underscores the need for designing a cost-effective nutrition intervention that can meet RDIs for the identified problem nutrients in the population.

In the present study, a mixture of whole grains flour and Irish potatoes (33 g), pulses and seeds (47 g), dried sardines and other animal source foods (5.6 g) and vitamin A rich fruits and vegetables (228 g) achieved the RDIs for 20 selected nutrients at a cost of 1122 Tanzanian shillings per day (TZS/day) (approximately 0.6 USD/day), which doubles the cost of the observed average food cost per day for a child aged between 6–8 months. Similarly, a mixture of whole grains flour (61 g), pulses and seeds (41 g), dried sardines and other animal source foods (36 g) and vitamin A rich fruits and vegetables (224 g) achieved the RDIs for 20 selected nutrients at a cost of 1335 TZS/day (approximately 0.7 USD/day), which is approximately twice the observed cost of food purchase per day for a child aged 9–11 months old. Likewise, a mixture of whole grains flour (85 g), pulses and seeds (44 g), dried sardines and other animal source foods (59 g) and vitamin A rich fruits and vegetables (348 g) achieved the daily intake recommendations for all selected nutrients at a cost of 2650 TZS/day (approximately 1.3 USD/day), which is a twofold increase of the observed food purchase cost for a child aged between 12 and 23 months. These results suggest that the alternatives for improving dietary adequacy of limiting nutrients are available but at a relatively higher cost than observed cost across all age groups. The increase in cost may affect the feasibility of optimal formulations in a population, especially in poor families. These findings propose the need for assessing diet patterns in other seasons as well as in other areas to identify other local, low-cost, nutrient-dense foods that can overcome the identified micronutrient gaps at an affordable cost for the studied population.

As in similar studies from other countries such as Indonesia, Myanmar and Cambodia, it was observed that essential micronutrients such as iron, zinc and calcium were the most difficult constraints to achieve given the existing complementary feeding practices [3,25,26,39]. Even though our modelled diets could not achieve DRIs for vitamin D, we did not regard this vitamin as a problem nutrient. This is because sufficient sun exposure could address the deficiency of vitamin D [40].

Our LP analysis showed that optimising the use of locally available nutrient-dense foods can improve recommended dietary intakes (RDIs) for nutrients like iron, zinc and calcium that are frequently reported as limiting nutrients among rural infants and young children in developing countries. The final optimal formulations of the present study contained a variety of food ingredients such as wholegrain cereals (maize flour, millet, rice, wheat), Irish potatoes, small dried fish (sardines), fruits (baobab powder), dried pulses, nuts and seeds (soybeans, peanuts, sesame seeds) and vegetables that can be sourced locally in the community. Also, final formulations contained a mixture of ingredients that can naturally increase the bioavailability of iron, zinc and vitamin A which are usually in a non-bioavailable form in most typical SSA diets [41,42]. The baobab powder in the mixture, for example, is a rich source of ascorbic acid, which is a well-known iron absorption enhancer [43,44]. Fish and poultry meat can increase the bioavailability of iron and zinc in the modelled formula [44]. In addition, sesame seeds in the mixture provide essential oils that can enhance the bioavailability of beta-carotene in the formulation [45]. The modelled diets contained iron and zinc absorption inhibitors such as phytates in whole grain cereals, seeds and legumes. Thus, experimental studies are needed to ascertain the biological value of essential nutrients in our optimal food formulations. Any future changes in the availability of local foods or price can be modified using the LP approach to generate ideal formulations that accommodate changes. Our goal is to ensure that the modelled formulations are available in areas with high prevalence of undernutrition.

Despite the identified strengths, this study has some limitations. The nutrient content of some candidate ingredients were missing from Tanzania’s Food Composition Tables, compelling us to use food composition databases from other countries. The content of nutrients in food is known to vary based on variety and location. This might have affected the results of our study, especially if the nutrient database of the modelled local foods in the final formulation was not accurate [3,46]. Also, we used median portion sizes above the 90th percentile for some identified potential foods as we believed that these foods could be successfully promoted. However, the feasibility of adopting these formulations may be difficult at the population level. Another limitation is that, given the range of the present study, our analysis did not take seasonality into account. As such, nutrient intakes and food consumption patterns identified refer basically to the dry season of the study area. Comprehensive studies, therefore, are needed to verify the effect of seasonality on food availability and consumption habits in the study population. Moreover, even though the models minimise deviations from the observed median sub-group patterns, the model will select the foods of highest nutrient content in each food sub-group. For this reason, other diets that conform to the median food sub-group patterns will not necessarily achieve the DRIs. These results mean there is one diet from amongst all possible diets that will achieve them.

In conclusion, our optimal food formulation derived from local foods achieved the nutritional goals for children aged 12–23 months at a twofold increase of the observed food budget per child. Also, the LP optimal formulations met the recommended intakes for 20 selected nutrients and achieved about 50% RDI for iron, which was identified as one of the problem nutrients among rural Tanzanian children aged 6–11 months. This implies that, given the available food choices, it is possible to develop optimal formulations that can improve micronutrient recommendations for rural children aged 6–23 months but at a cost which is relatively higher than the observed household budget. The findings from the present study suggest the need for setting interventions that can help the households to increase access to nutrient-dense foods that can overcome the identified micronutrient gaps in Tanzania.

## Supplementary Material

Observed_Food_Items_for_Children_Aged_6-23_Months__1_.xlsx
